# Paving the way to secondary dormancy: mind the *DOG*’s tail

**DOI:** 10.1093/plphys/kiaf008

**Published:** 2025-01-08

**Authors:** Dechang Cao

**Affiliations:** Germplasm Bank of Wild Species, State Key Laboratory of Plant Diversity and Specialty Crops & Yunnan Key Laboratory for Crop Wild Relatives Omics, Kunming Institute of Botany, Chinese Academy of Sciences, Kunming, Yunnan 650201, China; Assistant Features Editor, Plant Physiology, American Society of Plant Biologists, Rockville, MD 20855, USA

One of the most amazing things in the world may be that organisms, from tiny bacteria to large trees, can sense and respond appropriately to seasonal changes. Especially fascinating is that some seeds can even cycle back and forth between dormancy and nondormancy in response to seasonal changes. Such dormancy cycling usually happens in seeds with physiological dormancy, in which germination is prevented by the physiological status of the seeds, instead of by a water-impermeable seed coat (Baskin and Baskin 2014). During dormancy cycling, seeds may exhibit primary and secondary dormancy. Primary dormancy is an intrinsic ability to prohibit germination obtained by a seed at maturity. When the seed comes out of dormancy but does not germinate, it may reenter a dormant status known as secondary dormancy.

Recently, the *DELAY OF GERMINATION 1* (*DOG1*) gene has been recognized as a key player in regulating seed physiological dormancy. In *Arabidopsis thaliana*, defects in *DOG1* lead to the absence of primary dormancy (Bentsink et al. 2006). *DOG1* encodes a protein with unknown molecular function and is modified by alternative splicing in Arabidopsis. The full-length long form of *DOG1* (*lgDOG1*) consists of 3 exons, and the proximal splicing of the first 2 exons yields a short form *DOG1* (*shDOG1*) (Cyrek et al. 2016). *shDOG1* shows higher mRNA abundance than *lgDOG1* and is the major determinant of dormancy depth in Arabidopsis seeds. In some species, such as *Lepidium sativum*, the *DOG1* gene consists of the first 2 exons and only encodes the short form transcript (Graeber et al. 2014). The extensively demonstrated role of *DOG1* in seed dormancy of various species suggests a conserved mechanism of physiological dormancy (Nishiyama et al. 2021).

Expression of *DOG1* is highly regulated via various factors, including transcription factors, chromatin remodeling factors, and pre-mRNA 3′ end processing factors (Zha et al. 2019; Deng et al. 2023; Li et al. 2023). Moreover, *DOG1* is negatively regulated by an autonomous antisense noncoding RNA, namely *asDOG1*. *asDOG1* is transcribed from the 3′ end of *DOG1* in the antisense orientation and has been demonstrated to be a negative regulator of seed dormancy (Fedak et al. 2016). *DOG1* is sometimes regarded as a regulator of both primary and secondary dormancy, while a recent investigation showed that *DOG1* specifically determines the depth of primary dormancy but not the seasonal patterns of secondary dormancy in Arabidopsis (Footitt et al. 2020). Usually, secondary dormancy is observed to be related to primary dormancy; however, it has been reported that secondary dormancy can be induced in *Beta vulgaris* with the absence of primary dormancy (Hourston et al. 2022). Thus, the association and distinction between primary and secondary dormancy remains elusive.

In this issue of *Plant Physiology*, Wrona and colleagues (2024) suggest that chromatin remodeling machinery mediated by *BRAHMA* (*BRM*) regulates *DOG1* on the 3′ end to specifically tune secondary dormancy of Arabidopsis seeds. The authors found that Arabidopsis deficient in *BRM* showed enhanced secondary dormancy but maintained similar primary dormancy depth compared with wild-type seeds. BRM is a core subunit of the SWItch/Sucrose Non-Fermentable (SWI/SNF) chromatin remodeling complex, which has been reported to regulate gene expression by binding to the 3′ gene regions with TATA boxes (Archacki et al. 2016). The presence of TATA boxes in the 3′ region of *DOG1* suggests that it might be a target of BRM. The double mutant seeds deficient in both *BRM* and *DOG1* did not show secondary dormancy. Thus, it is very likely that *BRM* interacts with *DOG1* to regulate secondary dormancy in Arabidopsis.

In accordance with previous findings that the short isoform of *DOG1* is predominant and responsible for dormancy regulation of Arabidopsis seeds (Cyrek et al. 2016), the authors detected higher levels of *shDOG1* but not *lgDOG1* in *brm-3* compared with the wild-type seeds during the induction of secondary dormancy. A reduction of *DOG1* expression was detected in seeds after 4 h of imbibition, and the expression was restored during secondary dormancy induction. The restoration of *DOG1* levels during secondary dormancy induction was especially notable in the *brm-3* mutant, leading to dramatically higher levels than that of wild-type seeds. The enhanced expression of *shDOG1* in *brm-3* seeds during secondary dormancy induction may be responsible for the strengthened dormancy phenotype.

Further analyses of *brm-3*, as well as the double mutant *brm-3 dog1-4*, showed that BRM may play some pleiotropic effects on seed development. Metabolic profiling revealed substantial amounts of metabolites regulated by the synergistic action of *BRM* and *DOG1*. Expression of *BRM* especially affects glutathione levels in a *DOG1*-dependent manner, which may take a role in seed longevity. The authors performed 3′ RNA sequencing and found some shared differentially expressed genes between *brm-3* and *dog1-4* mutants compared with the wild type. More interestingly, the 3′ RNA sequencing of double mutant *brm-3 dog1-4* revealed that a substantial subset of *BRM*-affected genes are dependent on *DOG1* function.

Arabidopsis mutants deficient in some other components of the BRM-containing SWI/SNF complex (*3xbrd* and *swp73a*) also showed similar phenotypes of *brm-3*, including super reinduction of *DOG1* and enhanced secondary dormancy. It is highly likely that *BRM* regulates *DOG1* via the chromatin remodeling complex during secondary dormancy. Intriguingly, BRM is not able to regulate truncated *DOG1* with the 3′ end removed. Chromatin Immunoprecipitation-qPCR (ChIP-qPCR) experiments suggest that BRM binds to the *DOG1* gene mostly within exons 2 and 3 in nondormant seeds, while predominantly binding to exon 3 in secondary dormant seeds. This BRM binding region locates the promoter of *asDOG1*. These results suggest that *BRM* could tune expression levels of *DOG1* by regulating *asDOG1* during secondary dormancy induction.

To test if *BRM* regulates *asDOG1*, the authors generated *LUC* reporters driven by the *p_AS_DOG1* promoter. Significant downregulation of *LUC* transcripts was found in *brm-3* compared with the wild type, suggesting that *BRM* may directly regulate the *asDOG1* promoter activity. Another reporter system with *LUC* fused to *DOG1* mutated at the TATA box elements was also used for analyses. Mutation of the TATA box elements was previously reported to decrease *asDOG1* levels (Yatusevich et al. 2017). Consistently, the authors found a notable increase of the sense *DOG1* level and enhanced secondary dormancy in transgenic LUC-DOG1 lines carrying deltaTATA mutation, being similar to *brm-3* and *brm-5* mutants. Thus, it is very likely that *BRM* regulates *DOG1*-mediated secondary dormancy via *asDOG1*.

In several mutants deficient in the SWI/SNF complex, including *brm-3*, *3xbrd*, and *swp73a*, enhanced secondary dormancy was observed to parallel with decreased levels of *asDOG1*. It is likely that *BRM* regulated secondary dormancy via SWI/SNF-mediated chromatin remodeling. Changes of DNA accessibility at the *DOG1* locus were analyzed during the induction of secondary dormancy via the formaldehyde-assisted isolation of regulatory elements method. In dry seeds, *brm-3* showed similar patterns of DNA accessibility as the wild type, while the former showed considerably higher DNA accessibility at the end of exon 2 on the third day of secondary dormancy induction. The change was even more pronounced on the fifth day of dormancy induction, centering around the promoter region of *asDOG1*. These changes are in accordance with the ChIP-qPCR results that *BRM* binds to this area of *DOG1*.

Taken together, *BRM* is a new player of secondary seed dormancy. As a core subunit of the SWI/SNF complex, BRM binds to *DOG1* directly at the 3′ end (Fig. 1). During secondary dormancy induction, the BRM-containing SWI/SNF complex may change DNA accessibility of the *DOG1* 3′ end to regulate *asDOG1* transcription. Thus, *DOG1*, as a dual regulator, links primary and secondary dormancy in Arabidopsis, while the BRM-containing SWI/SNF complex acts on *DOG1* 3′ end to provide a specific regulatory machinery for secondary dormancy. Another report also noted that 3′ processing of *DOG1* pre-mRNA plays a role in seed dormancy of Arabidopsis (Li et al. 2023). Further exploration into the 3′ end of *DOG1* may help advance our understanding of secondary seed dormancy in the future.

**Figure 1.**
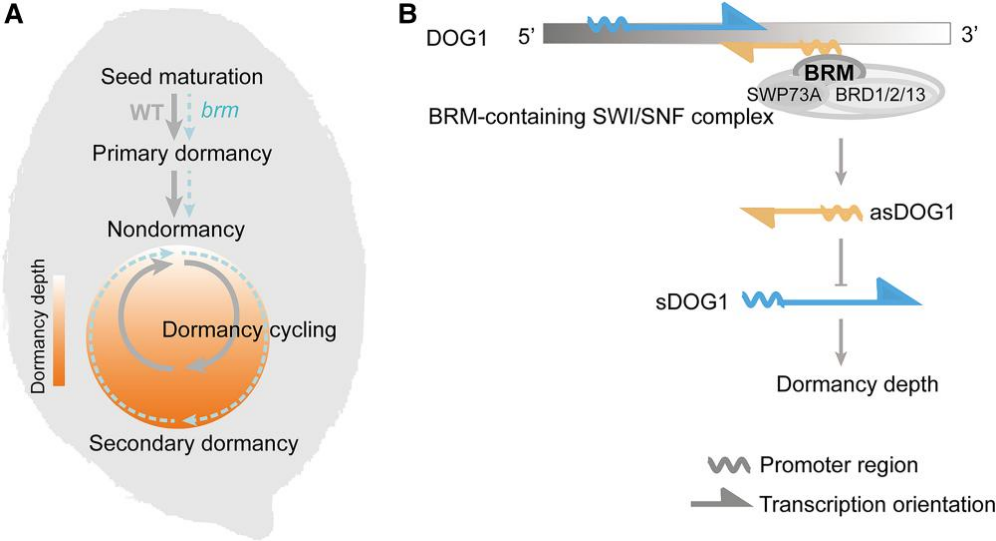
*BRM* specifically regulates secondary seed dormancy via targeting the *DOG1* 3′ end. **A)** A scheme describing dormancy cycling and the difference between primary and secondary dormancy. Arabidopsis mutants deficient in *BRM* showed enhanced secondary seed dormancy without affecting primary dormancy. **B)** A working model for *BRM* regulation of secondary dormancy in Arabidopsis seeds. BRM, with other subunits of the SWI/SNF complex, binds directly to the 3′ end of the *DOG1* locus on the chromatin. The binding of BRM-containing SWI/SNF complex increases DNA accessibility of the antisense *DOG1* (*asDOG1*) promoter region, thus promoting its transcription. Transcription of *asDOG1* negatively regulates expression of the sense *DOG1* (*sDOG1*), which is the determinant of dormancy depth. Thus, the BRM-containing SWI/SNF complex provides specific machinery to negatively regulate secondary dormancy via direct binding to the 3′ end of *DOG1*.

## Data Availability

No new data were generated or analyzed in support of this research.
